# Increased Serum and Musculotendinous Fibrogenic Proteins following Persistent Low-Grade Inflammation in a Rat Model of Long-Term Upper Extremity Overuse

**DOI:** 10.1371/journal.pone.0071875

**Published:** 2013-08-28

**Authors:** Helen G. L. Gao, Paul W. Fisher, Alex G. Lambi, Christine K. Wade, Ann E. Barr-Gillespie, Steven N. Popoff, Mary F. Barbe

**Affiliations:** 1 Department of Anatomy and Cell Biology, Temple University School of Medicine, Philadelphia, Pennsylvania, United States of America; 2 Department of Physical Therapy, Thomas Jefferson University, Philadelphia, Pennsylvania, United States of America; 3 College of Health Professions, Pacific University, Hillsboro, Oregon, United States of America; Johns Hopkins University School of Medicine, United States of America

## Abstract

We examined the relationship between grip strength declines and muscle-tendon responses induced by long-term performance of a high-repetition, low-force (HRLF) reaching task in rats. We hypothesized that grip strength declines would correlate with inflammation, fibrosis and degradation in flexor digitorum muscles and tendons. Grip strength declined after training, and further in weeks 18 and 24, in reach limbs of HRLF rats. Flexor digitorum tissues of reach limbs showed low-grade increases in inflammatory cytokines: IL-1β after training and in week 18, IL-1α in week 18, TNF-α and IL-6 after training and in week 24, and IL-10 in week 24, with greater increases in tendons than muscles. Similar cytokine increases were detected in serum with HRLF: IL-1α and IL-10 in week 18, and TNF-α and IL-6 in week 24. Grip strength correlated inversely with IL-6 in muscles, tendons and serum, and TNF-α in muscles and serum. Four fibrogenic proteins, TGFB1, CTGF, PDGFab and PDGFbb, and hydroxyproline, a marker of collagen synthesis, increased in serum in HRLF weeks 18 or 24, concomitant with epitendon thickening, increased muscle and tendon TGFB1 and CTGF. A collagenolytic gelatinase, MMP2, increased by week 18 in serum, tendons and muscles of HRLF rats. Grip strength correlated inversely with TGFB1 in muscles, tendons and serum; with CTGF-immunoreactive fibroblasts in tendons; and with MMP2 in tendons and serum. Thus, motor declines correlated with low-grade systemic and musculotendinous inflammation throughout task performance, and increased fibrogenic and degradative proteins with prolonged task performance. Serum TNF-α, IL-6, TGFB1, CTGF and MMP2 may serve as serum biomarkers of work-related musculoskeletal disorders, although further studies in humans are needed.

## Introduction

According to the Bureau of Labor Statistics report entitled Nonfatal Occupational Injuries and Illnesses Requiring Days Away from Work, 2011, musculoskeletal disorders accounted for 33 percent of all lost work time workplace injuries and illnesses in the U.S. and required a median of 11 days away from work [1]. Studies in humans with upper extremity work-related musculoskeletal disorders find evidence of inflammation, fibrosis and degeneration in serum and musculotendinous tissues, changes thought to induce concurrent motor dysfunction [2]–[8]. However, the pathophysiological responses are still under investigation, particularly responses associated with chronic myopathies and tendinopathies, as are serum biomarkers that might aid in pinpointing the stage of these disorders.

An inflammatory response in musculoskeletal tissues has been considered an important element in the pathogenesis of upper extremity soft tissue disorders [8]–[10]. A small number of studies have searched for and detected serum biomarkers of inflammation in patients with upper extremity musculoskeletal disorders of short duration (<3 months), including C-reactive protein, interleukin- 6 (IL-6), tumor necrosis factor-alpha (TNF-α), and members of the IL-1 family [2], [3], [4]. The results of these studies suggest a role for inflammatory cytokines early in the course of upper extremity MSDs. However, tissues collected from patients with upper extremity MSDs at the time of surgical intervention show increased IL-1β immunoreactive fibroblasts and IL-6 (which can be pro- or anti-inflammatory depending on accompanying cytokines) [11]–[13], but few acute inflammatory responses [7], [11]. Interestingly, IL-6, IL-1β and TNF-α have also been deemed as pro-fibrotic cytokines due to their mitogenic and chemotactic effects on fibroblasts and induction of fibrogenic proteins [14]–[19].

A few studies examining serum of workers have also detected increased serum biomarkers of collagen turnover in response to prolonged exposure to heavy physical loads. Increased serum markers of collagen type I synthesis (PINP; N-terminal propeptide type I procollagen) and degradation (CTX1; C-telopeptide of type I collagen) were identified in workers employed in heavy manual lifting jobs [20]–[22], although the overall ratio of these synthesis to degradation markers remained the same in male construction workers as in workers with sedentary jobs. These results indicate that stressed tissues can adapt to the needs of a particular job, increasing collagen synthesis to match that of collagen degradation. However, studies examining tendosynovial tissues collected from patients with upper extremity musculoskeletal disorders during surgical intervention show increased tissue fibrogenic and degradative proteins (e.g., transforming growth factor beta 1 and matrix metalloproteases) and fibrotic histopathology [7], [11], [23], [24], [25]. These latter findings are indicative of deranged extracellular matrix production and degeneration in tissues by the time of surgical intervention, rather than tissue adaptation.

Transforming growth factor beta 1 (TGFB1) and connective tissue growth factor (CTGF/CCN2) are important mediators of fibrosis. TGFB1 has been implicated as a sensitive serum biomarker of fibrogenic tissues changes [26]. Levels of CTGF/CCN2 in patients with scleroderma or other fibrotic disorders correlate positively with disease severity [27], [28]. Both are key mediators of tissue remodeling and fibrosis that work synergistically to generate sustained fibrosis [29], [30]. Serum matrix metalloproteases (MMPs) have also been indicated as sensitive serum biomarkers of pathological collagen remodeling occurring with osteoarthritis, ankylosing spondylitis, heart failure due to deranged myocardial collagen turnover, and a history of Achilles tendon rupture [31], [32], [33], [34]. One long-term goal of our lab is to identify biomarkers for monitoring disease progression of work-related musculoskeletal disorders, and for appropriate targeting of treatments [35], [36]. This is especially significant since fibrotic tissue changes, once present, are difficult to treat [37], [38], [39], [40]. However, neither TGFB1 nor CTGF have been examined in serum in association with work-related musculoskeletal disorders. Since CTGF is normally low or undetectable in sera of healthy individuals, it may serve as a predictive biomarker for patients in the fibrotic stage of work-related musculoskeletal disorders.

We have developed a rat model of voluntary reaching and grasping, in which reach rates and force levels were determined from epidemiological studies for risk exposure in humans [41]. We have observed exposure-dependent (level of task and time of exposure) motor declines. Performance of a high repetition low force, food retrieval, task for 8 weeks induced inflammatory cytokine increases and grip strength declines, while performance of a low repetition low force task did not [42], [43], [44], [45]. A follow up study in rats performing a high repetition low force (HRLF), handle-pulling, task for 12 weeks showed persistent grip strength declines despite resolution of an earlier pro-inflammatory cytokine response in serum [46]. In recent studies examining the effects of performance of a high repetition high force, handle-pulling, task for 6–12 weeks, we observed increased production of TGFB1 and CTGF in forelimb nerves, muscles and tendons [47], [48]. A two-week treatment of HRHF rats treated in weeks 5–6 of task exposure with ibuprofen or anti-TNF-α attenuated increased inflammatory cytokines, CTGF and fibrosis in forelimb muscles, and grip strength declines [47], [49]. From these results, we hypothesize that both pro-inflammatory responses and fibrotic responses contribute to motor declines, although their temporal pattern of expression may differ, with acute inflammatory responses appearing early and fibrotic responses appearing later with prolonged task performance. However, we have not examined the effects of performing repetitive tasks beyond 12 weeks in this model; this is necessary in order to parse out which mechanism, inflammation or fibrosis (or both), is contributing to persistent motor declines.

In this study, we extended our past shorter-term studies of 12 weeks or less, to examine the effects of performing the high repetition low force (HRLF), handle-pulling, task for 18 to 24 weeks on forearm grip strength, and on inflammatory, fibrotic or degradative responses in forearm muscle and tendons. We hypothesize that acute inflammatory responses contribute to early declines in grip strength and that fibrogenic tissue responses contribute to chronic grip strength declines. We also sought to identify serum biomarkers indicative of underlying tissue processes, hypothesizing that at least one serum biomarker of each process would be detectable in serum. To meet these goals, we assessed the following in young adult rats performing a HRLF handle-pulling task for 18–24 weeks: 1) forearm grip strength; 2) flexor digitorum muscles and tendons for mediators of inflammation, fibrosis and degradative changes; and 3) serum for similar protein analytes.

Adult female rats were used for several reasons: (1) Human females have a higher incidence of work-related musculoskeletal disorders than males [50]; (2) for comparison to data from our many past studies on female rats; (3) these rats were part of other studies examining the other tissues, such as bone and brains, that required use of female rats for consistency.

## Methods

### Subjects

**Figure 1:**
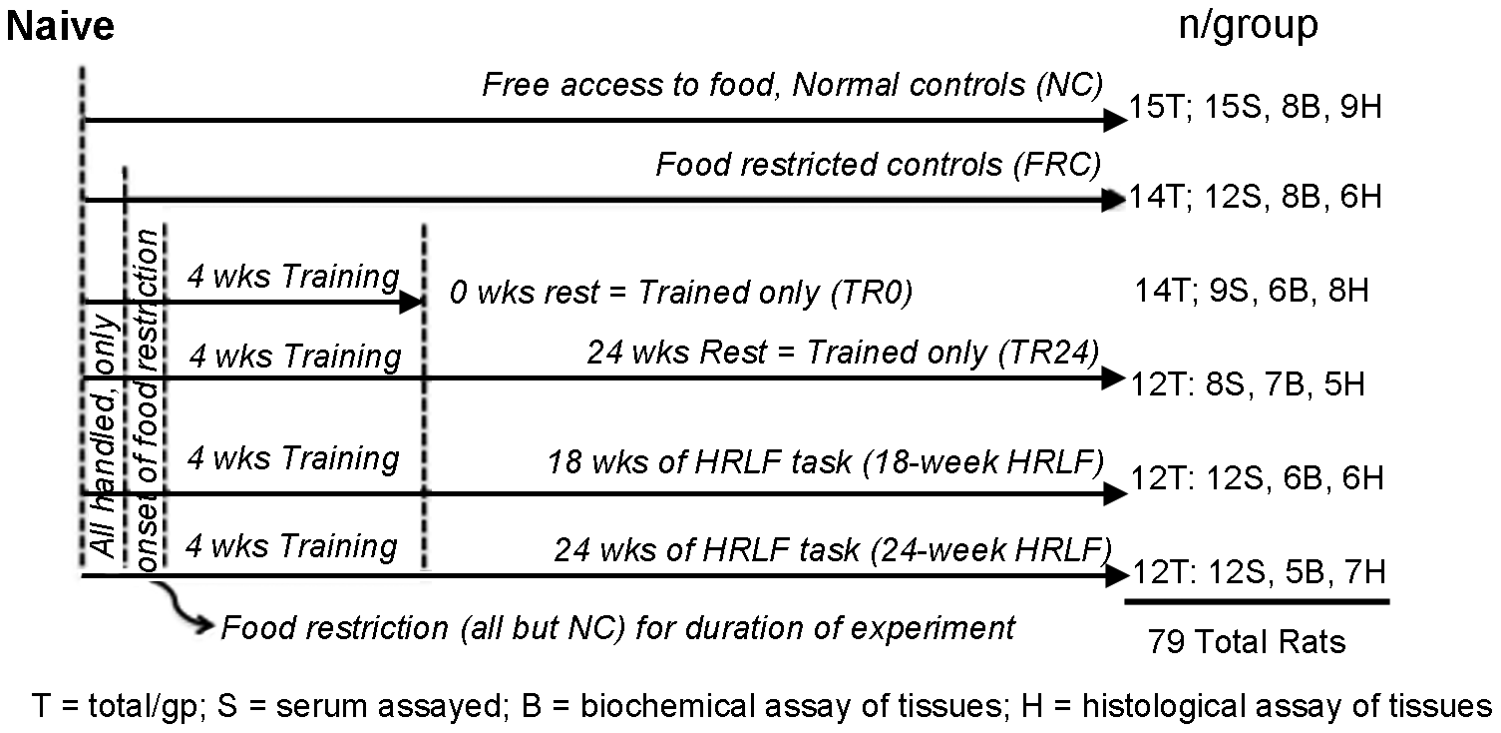
Design of experiment. All rats were handled daily for week 1. Then, all but normal control rats (NC) were food restricted to within 5% of the weights of the NC rats for the remainder of the experiments. Food restricted control rats (FRC) did not undergo training and did not perform the high repetition low force (HRLF) task. A cohort of rats were trained only for 4 weeks, and then euthanized (TR0). Another cohort was trained only for 4 weeks, and then rested for 24 weeks (TR24). Two groups of rats performed the HRLF task for either 18 weeks or 24 weeks (18-week HRLF and 24-week HRLF rats). After euthanasia, serum (S) was collected from all rats and assayed in nearly all rats. Half of the tissues were analyzed using biochemical methods (B) or histological methods (H).

The Temple University Institutional Animal Care and Use Committee approved all experiments in compliance with NIH guidelines for the care and use of laboratory animals. Seventy-nine total young adult, female Sprague-Dawley rats (3 mo. of age at onset of experiments) were used. As shown in Figure 1, rats were randomly divided into one of 5 groups: age-matched normal controls (NC); age- and weight-matched food restricted controls (FRC), age-matched trained-only rats that underwent the initial training, and that were then either euthanized after the training period (TR0), or rested for 24 weeks before euthanasia (TR24; TR+REST); and rats that were also trained to learn the task, and then performed the high repetition low force task (HRLF) task for 18 or 24 weeks before euthanasia (numbers/group are shown in Figure 1). Rats were housed in a central animal facility in separate cages with a 12-hour light: dark cycle and free access to water. Rats were weighed weekly and their food was adjusted to maintain ±95% body weight of age-matched controls in order to avoid catabolic tissue changes that might occur with greater weight loss, and to avoid confounds of obesity (the rats tend to work hard for the banana-flavored food pellets used as part of the reward for successful task performance). All rats were inspected weekly and again post-mortem for presence of illness or tumors in order to reduce confounders for serum cytokine increases (none were observed). To further reduce illness related confounders, additional sentinel rats were examined for presence of viral infections or other illnesses as part of the regular veterinary care (none were detected).

### Training Period

**Figure 2:**
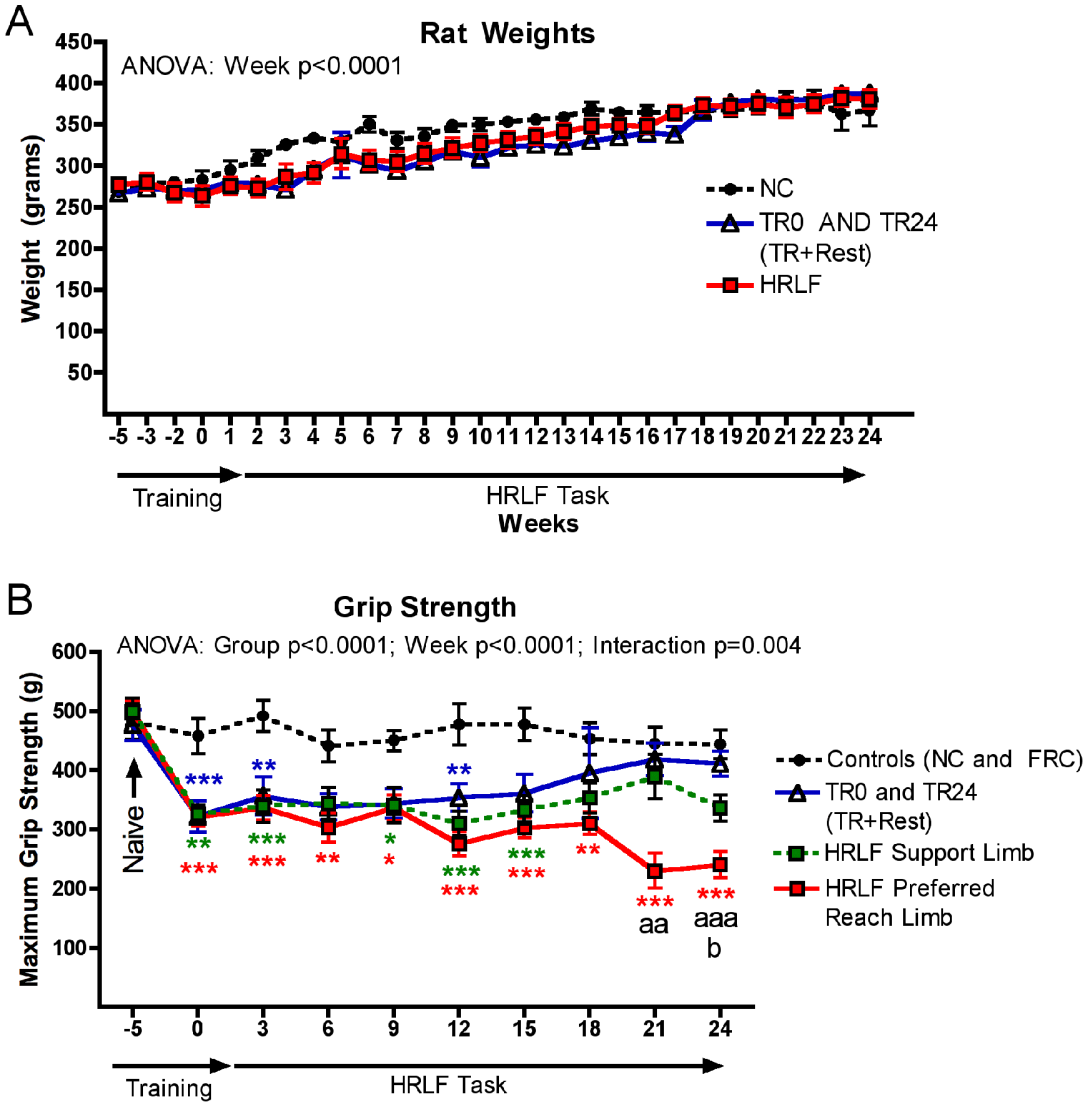
Weight and grip strength changes across weeks of task performance. (A) All rats gained weight across the 29 weeks. There were no significant differences in body weights between trained only (TR0 and TR24) or high repetition low force (HRLF) rats, compared to normal controls (NC) rats. (B) Maximum reflexive grip strength is shown for control rats (normal and food-restricted controls were combined as there were no significant differences between these groups), TR24 (TR+Rest; trained rats that rested after training for 24 weeks), and HRLF rats. The preferred reach limbs and the contralateral support limbs were analyzed separately in HRLF rats. The week 0 time point of each group is after the training period and before the HRLF task performance or the rest period. Symbols: *:p<0.05 and ***:p<0.001, compared to age-matched control rats; ^aa^: p<0.01 and ^aaa^:p<0.001, compared to age-matched TR24 rats; ^b^: p<0.05, compared to the support limb of HRLF rats.

Prior to the initiation of the experiments, all rats were handled for 10 minutes/day for 2 weeks. All but normal control rats were randomly selected and food-restricted for a short period (no more than 7 days) by 5–15% of their naive weight (i.e., they lost no more than 5–15% of naive body weight) to initiate interest in the food pellets (Fig. 2A). After that first week of food restriction, all food-restricted rats were given extra food chow and then maintained thereafter as closely as possible to within 5% of their naive weight until euthanasia (Fig. 2A). It is our experience that female rats require little food restriction for motivation after they have learned the task. Fourteen of the food-restricted rats did not proceed to training, and served as FRC rats (randomly selected; Fig. 1). Fifty more of the food-restricted rats went through an initial training period of 10–15 minutes/day, 5 days/week, for approximately 4 weeks, in which they were trained to perform the reaching and handle pulling task at a high repetition low force level. During this period, the rats moved through several stages of training. First, they were placed in an operant behavior box with a portal modified with an attached trough, and introduced to a 1∶1 mix of grain-based and banana-flavored pellets that served as food reward (Bioserve, Frenchtown, NJ). When they learned to reach (without a specified reach rate) into a trough for the food pellets, a time period of typically 3–7 days, they were moved to the custom-designed operant conditioning chambers, described in detail previously [51], and as depicted previously [48]. In the chambers, rats learned with the aid of auditory and light cueing to reach through the portal, grasp the force handle, and exert an isometric pull on the force handle of at first approximately 1% and then 5% of their maximum voluntary force (the latter equal to 0.10 Newtons) without any specified repetition rate (1–2 weeks), and then at 0.23 Newton (15% of their maximum voluntary force, making this a low force task) without any specified repetition rate (another 1–2 weeks). By the end of this 4 week training period, the rats were able to perform the HRLF task of four reaches/min at 15% of their maximum voluntary force. A subset of these rats were randomly selected to serve as trained only rats (n = 26; Fig. 1), and did not proceed to HRLF task performance. Fourteen of these TR only rats were euthanized immediately after training in order to determine the immediate effects of training (TR0); twelve more rested for 24 weeks before euthanasia (TR24), while receiving a diet similar to the HRLF rats, in order to serve as age-matched, diet-matched training controls.

### HRLF Repetitive Task Regimen

Following the training period, subsets of trained rats were randomly chosen from the trained groups to become HRLF rats (n = 24; Fig. 1). The HRLF rats reached and pulled a handle at a rate of 4 reaches/minute at 15±5% of maximum voluntary force, for 2 hours/day in 30 minute sessions, for 3 days/week, for up to 24 weeks.. The task was divided into four, 0.5-hour sessions separated by 1.5 hour in order to avoid satiation. Because the inherent nature of our task is voluntary, the rats tended to over-reach, maintaining an average 5.04±0.45 successful reaches/minute rather than the target rate of four reaches/minute. In addition, they were not prevented from reaching at a higher or lower force than the target of 0.23 Newtons (15% of maximum pulling force). However, a food reward was not given unless they met the force criterion within a 5 sec window initiated every 15 sec. Limb dominance was not evident until after the training period, but was evident during the task regimen, and was recording thereafter. Rats were allowed to use their preferred limb to reach (the “reach” limb), and their contralateral limb as support against the operant chamber wall while pulling (the “support” limb), as described and depicted previously [48]. Thus, the animals were allowed to self-regulate their participation in task performance, making this an operant, voluntary task.

### Motor function assay

Grip strength was measured in all animals bilaterally at baseline, after training, and every 3 weeks thereafter, using a rat grip strength recording unit (Stoelting, Wood Dale, IL), as described previously [51], by an examiner naïve to group assignment (CKW). The test was repeated 3–5 times/limb/trial, and maximum grip strength per trial is reported.

### Serum assays

Following euthanasia (Nembutal, 120 mg/kg body weight), 36 hours after completion of the final task session, blood was collected from all rats by cardiac puncture using a 23-gauge needle and centrifuged immediately at 1000 g for 20 min at 4°C. Serum was collected and stored at −80°C until analyzed, and then assessed for 13 analytes in 8–15/group, as shown in Figure 1. Using customized multiplex ELISA plates (Aushon Biosystems, Billerica, MA), serum was assessed for: interleukin (IL)-1α, IL-1β, IL-2, IL-4, IL-6, IL-10, macrophage inflammatory protein (MIP) 2 and MIP3; matrix metalloproteinase 2 (MMP2), platelet derived growth factor ab and bb (PDGFab and PDGFbb), and tumor necrosis factor-alpha (TNF-α). Serum was also tested using single-plex ELISA kits for CTGF, IL-12, PINP (N-terminal propeptide type I procollagen), hydroxyproline, TGFB1 (kits sources were: rat CTGF from Bio Medical Assay, Beijing, China, #22202; IL-12 from Invitrogen, Grand Island, NY, # KRC0121; rat/mouse PINP from immunodiagnostics systems, Scottsdale, AZ, #AC33F1; Hydroxyproline Assay Kit, MAK008, Sigma-Aldrich, Saint Louis, MO; and rat TGFB1 from Aushon Biosystems). All serum samples were analyzed in duplicate, according to manufacturer's directions, and presented as pg/ml or micrograms/ml of serum.

### Tissue biochemical assays

Following euthanasia and serum collection, rats were divided into two approximately equal subgroups, one for biochemical analysis of the flexor digitorum muscle and tendons (5–8/group) and another group for histological and immunohistochemical analysis (n = 5–9/group), as shown in Figure 1. The muscle and tendons for biochemical assay were collected from the distal forearm region, bilaterally (preferred reach and support limb tissues were collected), as previously described [48], and flash frozen in liquid nitrogen. For each tissue type, the outer and inner connective tissue sheaths were collected with the internal myofibers or endotendon regions included for muscle and tendons, respectively. These tissues were homogenized in RIPA buffer using previously described methods [42]. Supernatants of the tissue homogenates were stored at −80°C, until assayed using ELISA kits and manufacturers' instructions for 11 analytes: IL-1α, IL-1β, IL-6, IL-10, IL-12, MIP2 and TNF-α using single ELISA kits, (each from Invitrogen), and for CTGF, TGFB1, PDGFab, PDGFbb, hydroxyproline, and MMP2, using the same ELISA kits as for serum assays (serum and tissues were batched assayed with kits from Bio Medical Assay or Aushon Biosystems). Each sample was run in duplicate. ELISA assay data (pg cytokine protein) were normalized to µg of total protein, determined using a bicinchoninic acid protein assay kit (Thermo Scientific, Pierce, Rockland, IL, #23225).

In addition to the ELISA assays, subsets of samples (n = 3–4/group) were examined using western blot or gelatin zymogram methods. TGFB1 Western blots were performed on supernatants from homogenized flexor digitorum muscles from NC (n = 4), TR0 (n = 4), 18-week HRLF (n = 4), and 24-week HRLF (n = 3) rats. 100 µg of protein was loaded per lane, with reducing agent, on NuPage Novex Bis-Tris mini gels. Anti-rat TGFB1 antibody was used (1∶2000; Santa Cruz Biotechnology, Inc., Dallas, TX, #sc-146), followed by probing with a secondary antibody tagged with IRDye800CW (1∶10,000; Li-Cor, #.926-32211), and then Li-Cor Odyssey Infrared Imaging System. Blots were also probed with GAPDH (1∶2000; glyceraldehyde-3-phosphate dehydrogenase; Invitrogen, #AM4300), and a secondary antibody tagged IRDye680LT (1∶10,000; Li-Cor, #926-68020), for use as a loading control. Western blots were repeated three times.

Gelatin zymogram gels were used to examine MMP2 and MMP9 presence and activity in homogenized flexor digitorum muscles from NC (n = 4) and 24-week HRLF (n = 3) rats. Supernatants of muscle homogenates were purified with gelatin Sepharose 4B beads (GE Healthcare Life Science, Pittsburgh, PA, #17-0956-01) by adding 500 µg total protein lysate (in 200 µl PBS) to 25 µl of a 50% slurry of washed gelatin Sepharose 4B beads in PBS, and incubating them on a shaker for 2 hr at 4°C. Beads were centrifuged at 2000 rpm for 1 min in a microfuge and washed with PBS; PBS was aspirated off with a small bore needle. Beads were resuspended in 25 µl of 2× load buffer, warmed to 50°C for 5 min, and 20 µl loaded per lane onto 10% zymogram gels (Novex, Life Technologies, #EC61752BOX) using manufacturer's instructions. Purified recombinant rat MMP9 and homogenized mouse spleen (gifts from Janssen R&D, Springhouse, PA) were used as positive controls. Gels were washed with 1× renaturing buffer, then stained with SimplyBlue Safe Stain (Life Technologies, Grand Island, NY, #LC6060), and imaged with an ImageQuant LAS 4000 (GE Healthcare Life Science). The location of gelatinolytic activity was detectable as a clear band against a background of uniform staining.

### Immunohistochemical Analyses

The remaining animals were used for immunohistochemical analysis to localize protein expression within the tissues of 5–9/group, as shown in Figure 1. Following euthanasia and after serum collection, animals were perfused transcardially with 4% buffered paraformaldehyde. Forearm musculotendinous tissues were collected *en bloc* (i.e. endotendon with attached epitendon, and myofibers with attached epimyseum) and sectioned longitudinally, as described and depicted previously [43], [48]. Sections were immunostained for CTGF and TGFB1 (anti-CTGF, 1∶400 dilution in PBS; anti-TGFB1, 1∶300), using previously described methods [47]. Western blot analysis was used to determine antibody specificity of the TGFB1 antibody; the specificity of the CTGF antibody used was published previously [47]. Negative control staining was also performed by omission of either primary or secondary antibodies, and by using tissues from normal control rats. Further negative control staining was performed by replacing the primary antibodies with an isotype matched IgG antibody, followed by the secondary antibody, in order to assay for any non-specific binding of the secondary antibodies (none were observed).

### Tendon Histopathology

A series of adjacent sections from above were stained with hematoxylin and eosin (H&E), dehydrated and coverslipped with DPX mounting medium. These sections, as well as the CTGF-immunostained sections, were examined for histopathological changes in tendons of NC (n = 5), TR24 (n = 5), 18-week HRLF rats (n = 5 each), and 24-week HRLF rats (n = 4). Both endotendon and epitendon regions of the flexor digitorum tendons were assessed for histopathological changes using a modification of the semi-quantitative Bonar scale method. We assessed only 3 factors, each on a 4 point (0–3) scale: cell shape, collagen organization, and vascularization, using previously described methods [48], [52]. Each of these determinations was made in the flexor digitorum tendons in the distal forearm to the wrist, in 3 microscope fields/section, and in 3 separate sections/rat, by one person naïve to group assignment (HG). We then used a computerized quantification system (Bioquant Osteo II) to quantify the epitendon thickness in the H&E stained sections (using the length array option of the system), and epitendon cellularity in the CTGF stained slides, by counting the number of CTGF-immunopositive cells per area (mm^2^), using previously described methods [53]. Again, these determinations were made in the flexor digitorum tendons in the distal forearm to the wrist, in 3 microscope fields/section, and in 3 separate sections/rat, by one person naïve to group assignment (MFB).

### Statistical Analyses

All data are expressed as mean ± SEM. Weight was analyzed using 2 way ANOVAs with the factors group and week, grip strength with the factors week and limb, and biochemical assays for inflammatory cytokines with the factors group and limb. Most other biochemical assays used one-way ANOVAs with group as the factor. In each statistical analysis, the Bonferroni method for correcting for multiple comparisons was used for the post-hoc analyses. Two-tailed t-tests were used to compare CTGF ELISA results (TR24 versus 18 week HRLF). For all analyses, adjusted p values are reported, and after adjustment, p values of <0.05 were considered significant. Pearson's correlation analyses were used to determine if grip strength values correlated with serum or tissue inflammatory cytokine levels, and if serum cytokines levels correlated with tissue cytokines levels; except for CTGF in which only n = 4 per group were tested and therefore, a Spearman's rank correlation test was used instead.

## Results

For all data, no differences were found between normal control (NC) and food restricted (FRC) rats; therefore, data from both groups were combined into a single control (C) group. Post hoc test results are shown in the figures for succinctness.

### Rat weights – all rats gained weight across the weeks

All rats gained body weight across the weeks of task performance (2 way ANOVA: Week: p<0.0001), which was important, since they were young adult rats at the onset of these experiments (Fig. 2A). The trained only rats (TR0 and TR24, which were the TR+Rest) rats did not have significantly lower body weights than the normal control (NC) or HRLF rats, nor did the HRLF rats have significantly lower body weights than the normal control (NC) rats. The food restricted control rats had similar body weights as the trained only and HRLF rats (data not shown).

### Grip Strength declined progressively in the HRLF reach limb, but recovered in the support limb

Forearm grip strength was significantly reduced, bilaterally, in trained and HRLF rats after the initial training period, compared to control rats (Fig. 2B; 2 way ANOVA: Group p<0.0001, Week: p<0.0001, Interaction p = 0.004; see figure for post hoc results). These declines persisted throughout this study and even worsened in preferred reach limbs of HRLF rats in weeks 18 and 24, compared to age-matched controls and TR24 (TR+Rest) rats. In contrast, grip strength declines present after training recovered to control levels in TR24 (TR+Rest) rats that rested for 24 weeks. A partial recovery was observed for grip in the contralateral support limb by week 18, compared to NC, and showed improvement, compared to preferred reach limbs of these same HRLF rats in week 24 (Fig. 2B).

### A mix of transient, cyclical and late-appearing inflammatory cytokines in musculotendinous tissues

**Figure 3:**
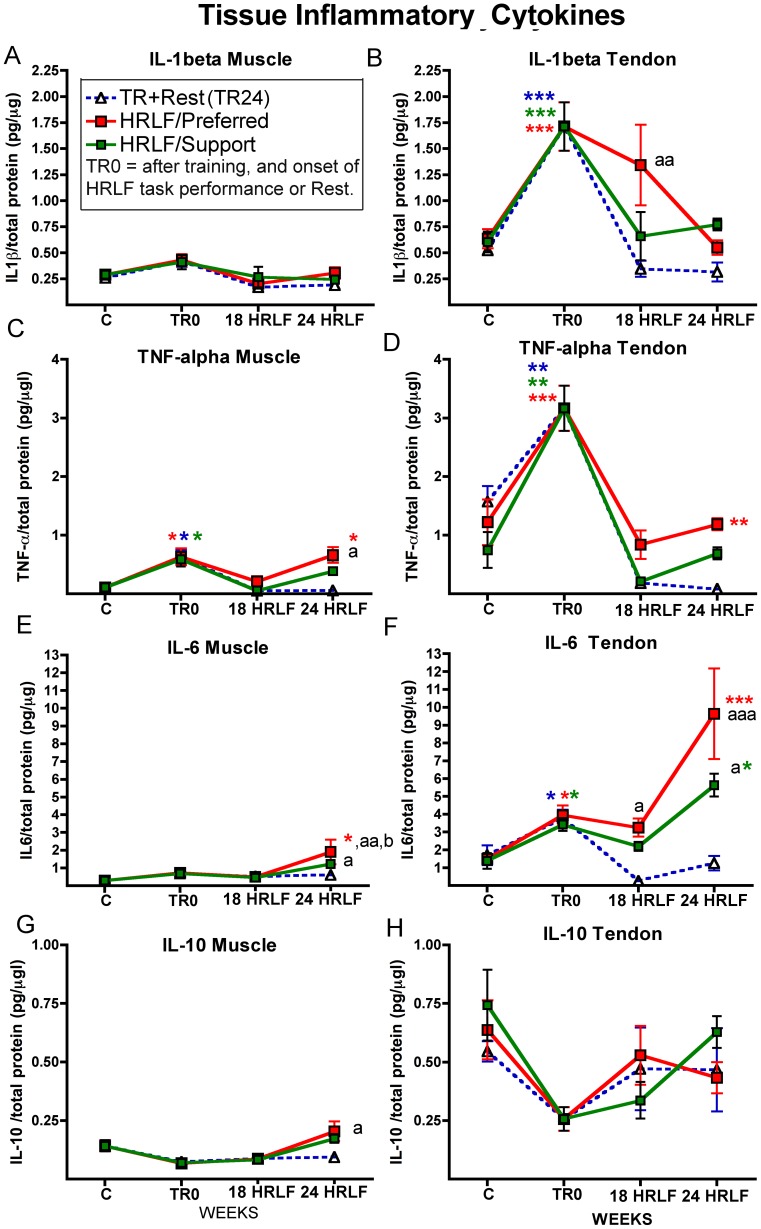
Pro- and anti-inflammatory cytokines in forelimb flexor digitorum tissues. Data shown for cytokines assayed in muscles and tendons after collection from control rats (indicated as C; normal and food restricted control data was combined as there was no significant difference), trained only rats euthanized immediately after training ( indicated as TR0 on the x-axes), TR+Rest (TR24; trained rats that rested for 24 weeks; blue dashed lines), and high repetition low force (HRLF) rats that worked for 18 or 24 weeks of task performance before tissue collection. The preferred reach limbs (red line) and the contralateral support limbs (green lines) were examined separately in HRLF rats. (A & B) Muscle and Tendon IL-1beta. (C & D) Muscle and Tendon TNF-alpha. (E & F) Muscle and Tendon IL-6. (G & H) Muscle and Tendon IL-10. Each analyte was assayed in duplicate using single-plex ELISA kits. Symbols: *:p<0.05, **:p<0.01 and ***:p<0.001, compared to age-matched control rats; ^a^: p<0.05 and ^aa^:p<0.01, compared to age-matched TR+Rest rats; ^b^: p<0.05, compared to the support limb of HRLF rats.

Since we have previously shown that grip strength declines correlate with increased muscle and tendon cytokines in rats performing a similar task for 8 weeks [44], we next examined flexor digitorum muscles and tendons, bilaterally, for inflammatory cytokines using ELISA (Fig. 3).

In the preferred reach limb, we found low-grade but significant increases in several pro- and anti-inflammatory cytokines in both tissue types after training and prolonged HRLF task performance, although greater increases were observed in tendons than in muscles, and greater in reach limbs than in the support limbs (Fig. 3). IL-1β was not increased in reach limb muscles with training or task performance (Fig. 3A). However, IL-1β was increased in reach limb tendons after training (TR0 time point) (Fig. 3B), compared to control (C) rats, and in 18-week HRLF reach limb tendons, compared to TR24 rats (Fig. 3B). TNF-α was increased after training, in reach limb muscles and tendons, resolved by week 18, but increased again in 24-week HRLF reach limb tissues, compared to C rats; and in 24-week HRLF reach limb muscles, compared to TR24 rats (Fig. 3C,D). IL-6 was increased in 24-week HRLF reach limb muscles, compared to C and TR24 rats, and compared to the support limb (Fig. 3E). IL-6 was also increased in reach limb tendons after training, but increased further in 24-week HRLF tendons, compared to C rats and TR24 rats (Fig. 3F). IL-10 was increased only in 24-week HRLF reach limb muscles, compared to TR24 rat muscles (Fig. 3G). Additionally, IL-1α was increased in reach limb muscles of 18-week HRLF rats (1.25±0.57, mean ± SEM), compared to C rats (0.12±0.03; p<0.01), and in reach limb tendons of 18-week HRLF rats (2.29±0.69), compared to C rats (0.30±0.04; p<0.01) (data not shown). No increases were seen for IL-12 or MIP2 in any group or tissue, compared to C rats (data not shown).

In the support limb, fewer changes were seen than in the reach limb tissues (Fig. 3). No increase in IL-1β was seen in support limb muscles (Fig. 3A). IL-1β was increased significantly in support limb tendons after training (TR0 time point), compared to control (C) rats (Fig. 3B), but resolved thereafter. TNF-α was increased after training in the support limb muscles and tendons, but showed resolution by week 18 (Fig. 3C,D). IL-6 was increased in 24-week HRLF support limb muscles, compared to TR24 rats, but showed no increase compared to control rats, and was lower than in reach limb muscles (Fig. 3E). IL-6 was also increased in support limb tendons after training, showed resolution by week 18, but increased again in 24-week HRLF rats, compared to C rats and TR24 rats (Fig. 3F). IL-10 was not increased in support limb tissues (Fig. 3G,F), nor was IL-1α (data not shown).

### Increased pro- and anti-inflammatory cytokines in serum with prolonged HRLF task performance

**Figure 4:**
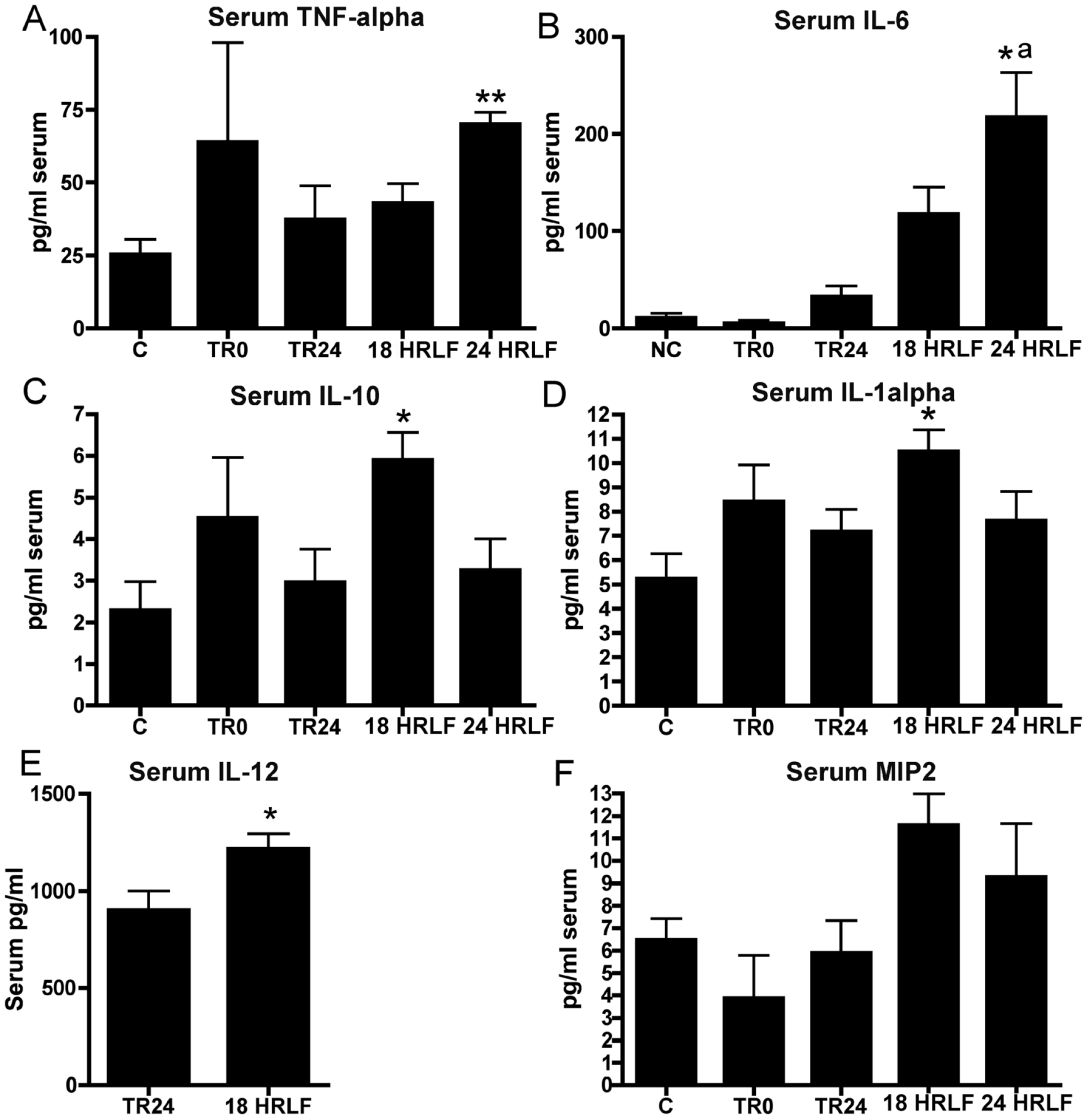
Pro- and anti-inflammatory cytokines in serum. Groups are as defined in figure legend 2. Data is shown for (A) TNF-alpha, (B) IL-6, (C) IL-10, (D), IL-1alpha, (E) IL-12 (only TR24 and 18 wk HRLF rat serum were tested for IL-12), and (F) MIP2. Symbols: *:p<0.05, **:p<0.01, compared to age-matched control rats. ^a^: p<0.05, compared to age-matched trained rats that rested for 24 weeks after the initial training period.

We next examined serum for these same and related cytokines to determine if there were concomitant increases in serum as in tissues. TNF-α and IL-6 were increased in serum of 24-week HRLF rats, compared to C rats (ANOVA results were p = 0.02 and 0.001, respectively; Fig. 4A,B). IL-6 was also higher in 24-week HRLF rats, compared to TR24 rats (Fig. 4B). IL-10, IL-1α and IL-12 were increased in serum of 18-week HRLF rats, compared to C rats (ANOVA results were p = 0.03, 0.03 and 0.02, respectively; Fig. 4C–E). Serum MIP2 levels trended higher in 18-week HRLF compared to C rats, but did not reach significance (ANOVA: p = 0.07; Fig. 4F). No serum increases were seen for IL-1β, IL-2, IL-4 or MIP3 (data not shown). There were also increases of TNF-α, IL-10, and IL-1α in the serum of TR0 rats, although there was high variability (and thus no significant increase).

### Increased fibrogenic proteins in serum and musculotendinous tissues with long-term task performance

**Figure 5:**
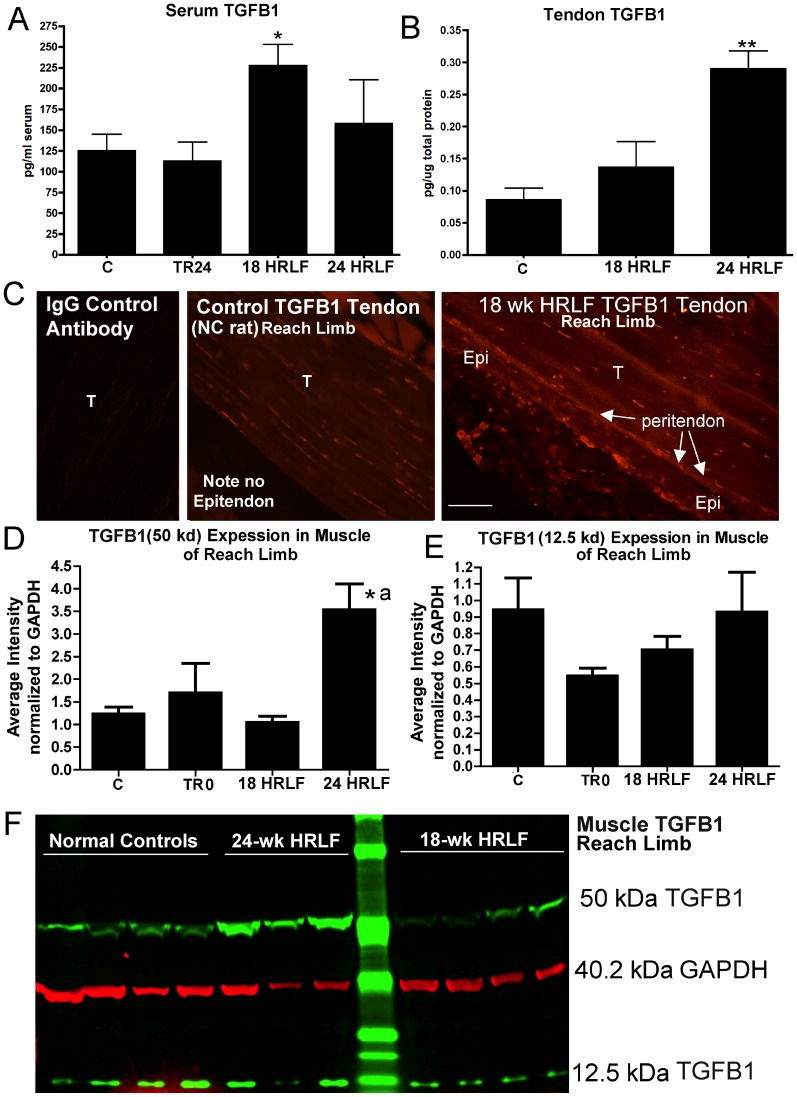
Transforming growth factor beta 1 (TGFB1) in serum and flexor digitorum tissues of the reach limbs. Groups are as defined in figure legend 2. (A&B) Serum TGFB1 and tendon TGFB1, assayed using ELISA. (C) Immunohistochemical staining for TGFB1 in tendons from NC and 18-week HRLF reach limbs shows localization of TGFB1 in fibroblast-like cells in the peritendon region of both the NC and HRLF rats, and additional stained cells in the epitendon (Epi; thickened in HRLF rats) and endotendon (T) regions of the HRLF rat tendon. (D&E) The results of Western blot analysis of muscle TGFB1 in which two bands were detected, 50 kDa and 12.5 kDa. The ratio of each band of TGFB1 normalized to GAPDH levels is shown for three replicates of the western blot. (F) A representative Western blot of reach limb muscles from normal controls (NC, n = 4 shown), 24-week HRLF rats (n = 3 shown), and 18-week HRLF rats (n = 4 shown), probed with anti-TGFB1 and GAPDH. Green bands were detected with an anti-TGFB1 antibody and a secondary antibody tagged with IRDye800CW (Li-Cor, #.926-32211). Red bands were detected with an anti-GAPDH antibody and a secondary antibody tagged IRDye680LT (Li-Cor, #926-68020). Symbols: *:p<0.05, **:p<0.01, compared to age-matched control rats; ^a^: p<0.05, compared to TR24 rats. Scale bar = 50 micrometers.

**Figure 6:**
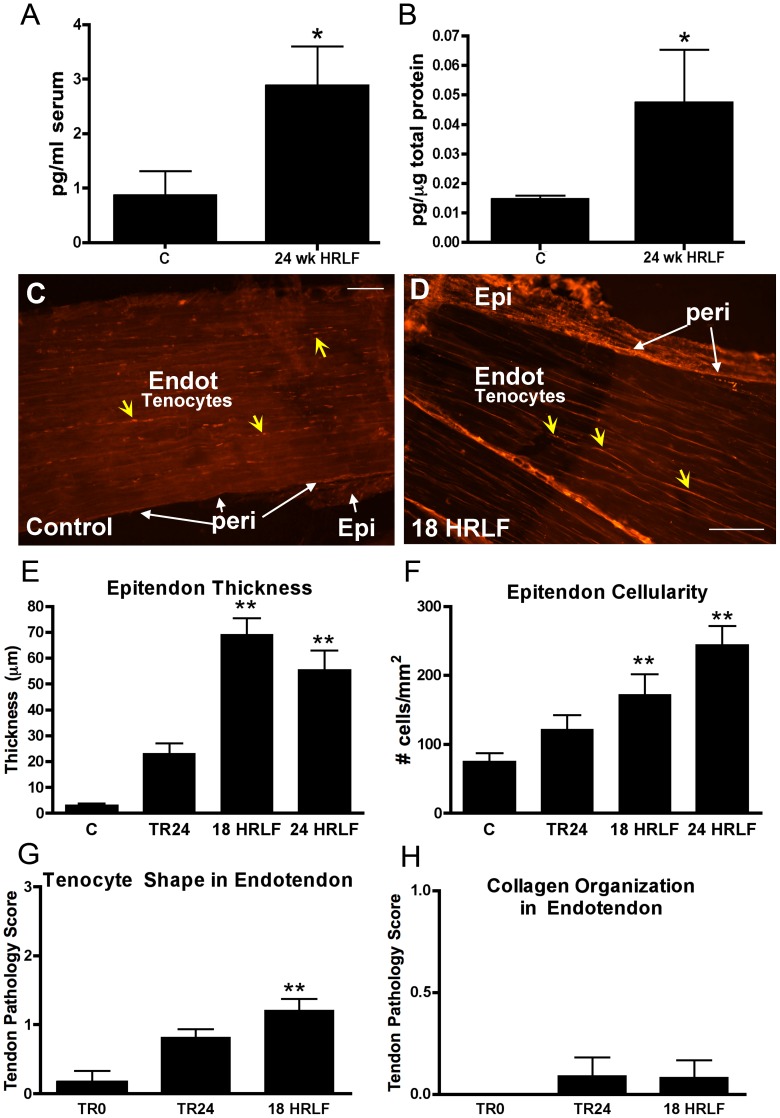
Connective tissue growth factor (CTGF) in serum and flexor digitorum tissues of the reach limbs. Groups are as defined in figure legend 2. (A) Serum CTGF and (B) muscle CTGF, assayed using ELISA. *:p<0.05, compared to age-matched control rats. (C&D) Immunohistochemical localization of CTGF in flexor digitorum tendons from control (normal control) and 18-week HRLF rats. Epitendon (Epi) is no longer distinct from peritendon (peri) regions in this HRLF rat. (E&F) Quantification of epitendon thickness and number of CTGF-immunopositive cells in the epitendon. *:p<0.05 and **:p<0.01, compared to age-matched control rats. Endot = endotendon. Scale bar = 50 micrometers.

**Figure 7:**
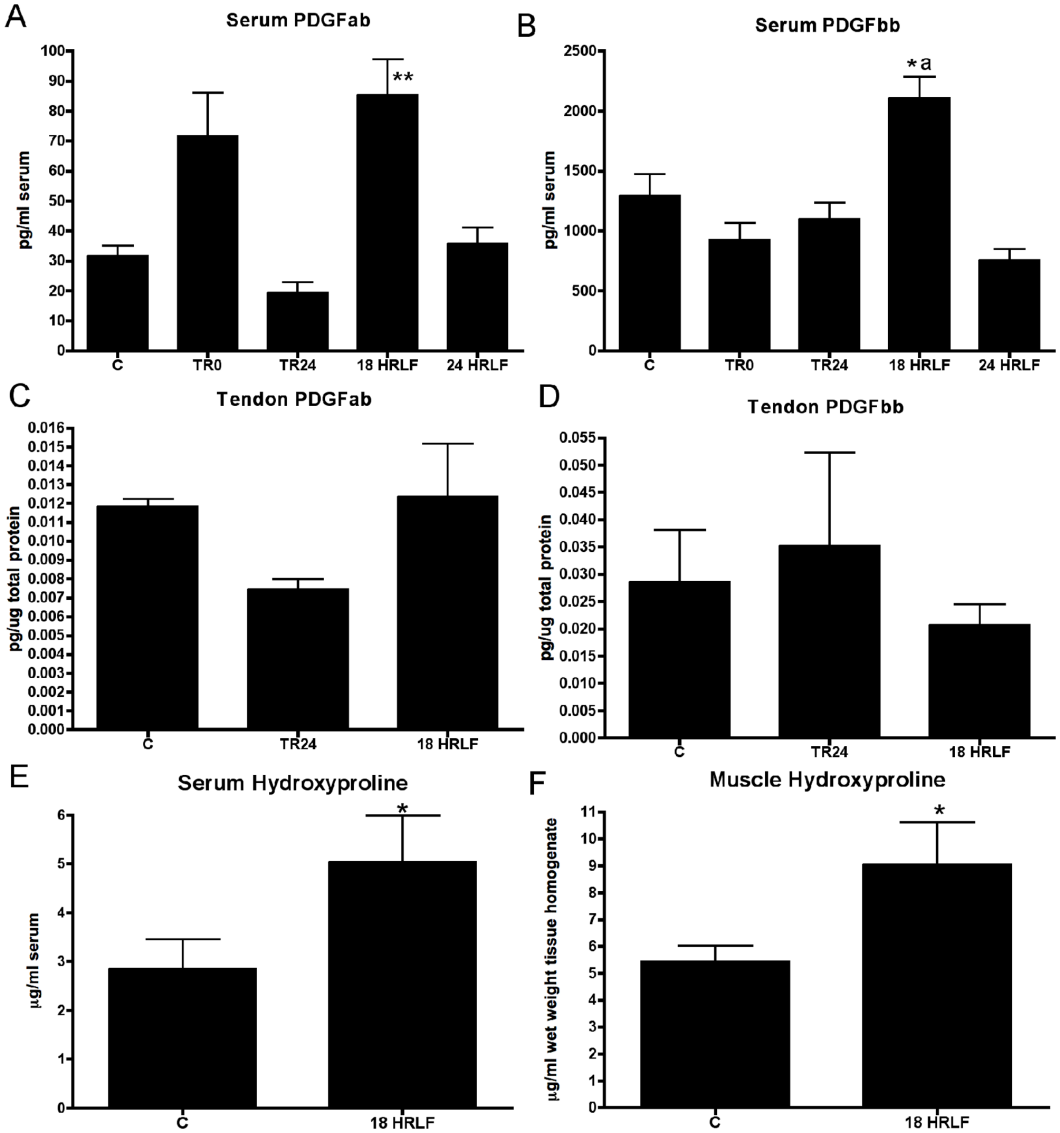
Platelet derived growth factor ab (PDGFab) and PDGF bb in serum and flexor digitorum tendons of the reach limbs, and hydroxyproline in serum and flexor digitorum muscles of the reach limbs. ELISA data for normal controls have been combined with that of food restricted controls (C); TR0 (trained only rats), TR21 (trained rats that rested for 21 weeks), and high repetition low force (HRLF) rats that performed the task for 18 or 24 weeks. ** and *: p<0.05 and p<0.01, compared to C rats; ^a^: p<0.05, compared to TR0 rats.

Since IL-6 and TNF-α are not only pro-inflammatory cytokines, but also pro-fibrogenic cytokines, we next extended our study to examine for increases of four other fibrogenic cytokines: TGFB1, CTGF, and PDGFab and PDGFbb (Figures 5–7). We focused on the reach limb tissues, since they showed consistent significant grip strength declines and inflammatory cytokine increases, from control levels.

TGFB1 was increased in both serum and reach limb tendons (entire distal tendon, including epi- and endotendon) of 18-week HRLF rats, compared to C rats (ANOVA: p = 0.03 and 0.003, respectively; Fig. 5A,B), as assayed using ELISA. Immunohistochemistry was used to localize TGFB1 expression in the reach limb tendons and showed a few TGFB1-immunoreactive fibroblast-like cells in the peritendon, as well as TGFB1-immunoreactive tenocytes in the endotendons NC rats (Fig. 5C, middle panel), but more TGFB1-immunoreactive cells in the epitendon region of 18- and 24-week HRLF rat tendons, in fibroblast-like cells (Fig. 5C right panel; 24-week data not shown). The tenocytes in the endotendon region of 28-week HRLF rats appeared more TGFB1-immunorective, but were not increased in number. Western blot analysis of reach limb muscle showed that the 50 kDa subunit (the precursor form) of TGFB1 protein increased in reach limb muscles of 24-week HRLF rats, compared to both C and TR24 rats (ANOVA: p = 0.002; Fig. 5D,F), although the 12.5 kDa subunit of TGFB1 (the active form) did not increase (Fig. 5E,F).

CTGF was increased in serum and reach limb flexor digitorum muscles of 24-week HRLF rats, compared to C rats (ANOVA: p = 0.03, each; Fig. 6A,B), as assayed using ELISA. Immunohistochemistry was then used to localize CTGF expression and showed a qualitative increase of CTGF immunopositive fibroblast-like cells in the epitendon, and in tenocytes in the endotendon 18 and 24 week HRLF reach limb tendons, compared to control (C) rats (Figure 6C,D. The 24-week HRLF data is not shown, but looked similar to 18-week HRLF tendons.

Both quantitative and semi-quantitative image analyses were performed on reach limb tendons. Quantitative image analysis showed increased thickness of the epitendon of reach limb tendons in 18- and 24-week HRLF rats, compared to Control (C) rats (ANOVA: p<0.0001; Fig. 6E), and increased numbers of CTGF-immunopositive cells in the epitendon of 18- and 24-week HRLF rats, compared to Control (C) rats (ANOVA: p = 0.0006; Fig. 6F), each indicative of epitendon hyperplasia. We also assayed the reach limb endotendon using a semi-quantitative Bonar scoring system of tendon pathology, and observed a small but significant change in tenocyte shape in 18-week HRLF rats, compared to TR0 rats (ANOVA: p = 0.001; Fig. 6G). They were still slender, but were thicker and more elongated than in control rat endotendons (See Figure 5C right panel and Figure 6D). No evidence of collagen disorganization was seen in the reach limb endotendon of 18-week HRLF rats, compared to TR24 or TR0 rats (ANOVA: p = n.s.; Fig. 6H). Assessment of the support limb tendons of 18-week and 24-week HRLF rats using the full Bonar scoring showed no significant changes from control rat histomorphometry (data not shown).

There was an increase in serum levels of PDGFab and PDGFbb in 18-week HRLF rats, compared to C rats (ANOVA: p = 0.0008 and p<0.0001, respectively; Fig. 7A,B). However, flexor digitorum tendons (reach limb only examined) showed no increase of either using ELISA (Fig. 7C,D), indicating that they were not the source of serum PDGF. We also sought to determine if there were changes in serum levels of a collagen synthesis marker (PINP) or a collagen turnover (hydroxyproline) as a result of task performance. PINP was not significantly increased in serum of any trained or HRLF rats, compared to control (C) rats (data not shown). In contrast, hydroxyproline levels were increased significantly in both serum and reach limb muscle tissues of 18-week HRLF rats, compared to controls (ANOVA: p = 0.03 and p = 0.01, respectively; Fig. 7E,F).

### Increased collagenolytic gelatinase, MMP2, in HRLF serum and tendons

**Figure 8:**
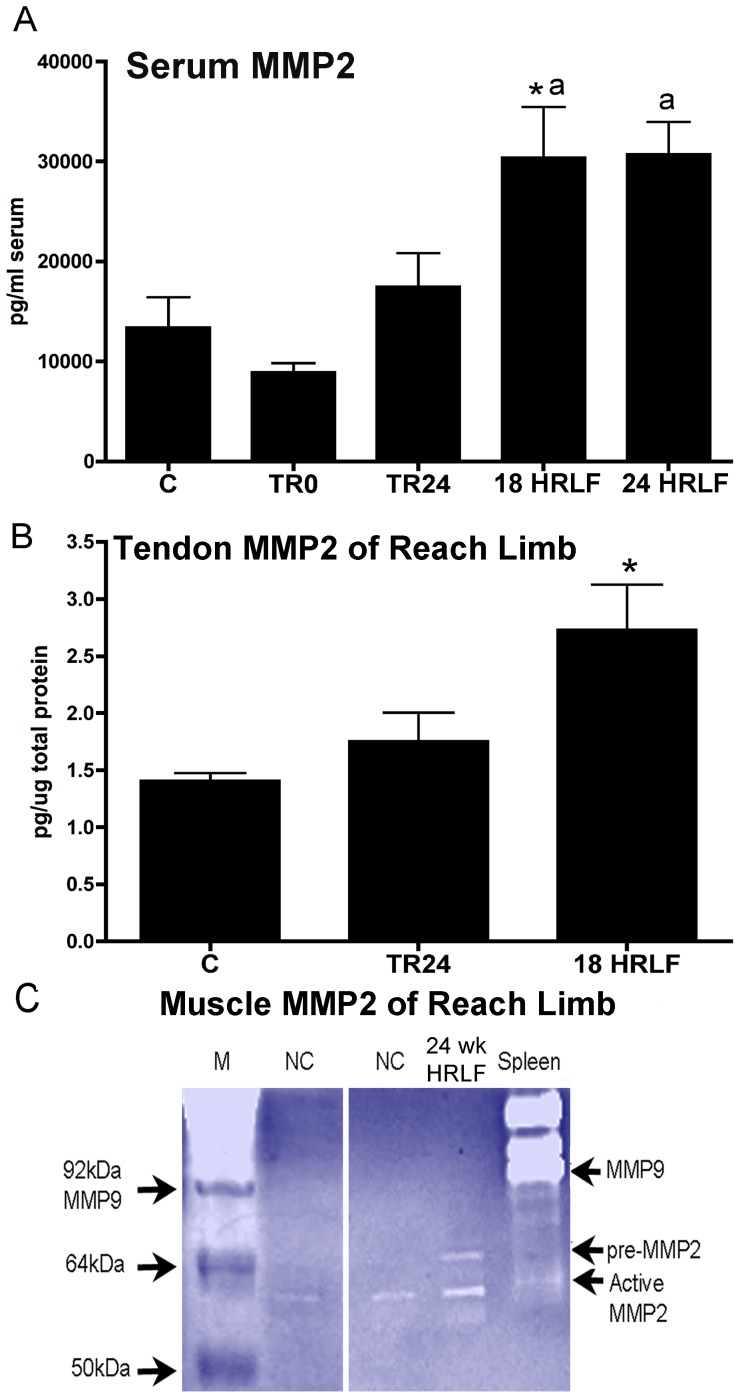
MMP2 levels in serum and flexor digitorum tendons and muscles of the reach limbs. Groups are as defined in figure legend 2. MMP2 levels in (A) serum and (B) flexor digitorum tendons tested using ELISA. *:p<0.05, compared to C; ^a^: p<0.05, compared to TR0 rats. (C) Gelatin zymography showing that muscle homogenates of 24-week HRLF rats have increased pro-MMP2 (72 kDa) and active MMP2 (62 kDa) compared to NC rats. Neither group showed any MMP9 activity. The far left lane is the marker (M). The far right lane was loaded with homogenized mouse spleen, which contains MMP9 and low levels of active MMP2.

The collagen degradation marker, MMP2, increased in serum of 18- and 24-week HRLF rats, compared to control (C) and TR0 rats (ANOVA: p = 0.04; Fig. 8A), and in reach limb tendons of 18-week HRLF rat weeks, compared to C rats (ANOVA: p = 0.04; Fig. 8B), each assayed using ELISA. The 24-week HRLF rat tendons from reach limb were not assayed for MMP2 due to insufficient quantities of tendon tissues. Gelatin zymography demonstrated that muscle homogenates of NC rats had low levels of active MMP2 (62 kDa subunit) but no increase of pro-MMP2 (72 kDa subunit). The 24-week HRLF rats had increases of both pro-MMP2 and active MMP2 in their reach limb muscles (Fig. 8C). No MMP9 was detected in the muscles, compared to mouse spleen homogenate (Fig. 8C) or purified recombinant rat MMP9 (not shown).

### Correlations between grip strength and cytokines/MMP2, and between serum and tissue analytes

As shown in Table 1, grip strength declines in the reach limbs correlated with increased IL-6, TNF-α and TGFB1 in flexor digitorum muscle from these same limbs; increased IL-6, TGFB1 and MMP2 in flexor digitorum tendons from these same limbs; and increased numbers of CTGF-immunopositive cells in the tendons from these same limbs. Grip strength declines also correlated significantly with increased serum IL-6, TNF-α, TGFB1 and MMP2, and showed a strong correlating trend with serum CTGF (p = 0.05). Increased serum IL-6, TNF-α, TGFB1, CTGF and MMP2 also correlated with increases of these same analytes in the tissues (Table 1).

**Table 1:** Significant Pearson's and Spearman's* Correlations.

Correlations with Grip Strength in reach limbs with muscle and tendon findings from same limbs			
	FD Muscle	FD Tendon	Serum
IL6	r = −0.49, p = 0.0004	r = −0.44, p = 0.0013	r = −0.30, p = 0.03
TNF-α	r = −0.32, p = 0.049	n.s.	r = −0.56, p = 0.0001
TGFB1	r = −0.59, p = 0.004	r = −0.71, p = 0.002	r = −0.59, p = 0.004
CTGF	n.s.*	r = −0.73, p = 0.0001#f	r = −0.50, n.s.
MMP2	n.t.	r = −0.62, p = 0.009	r = −0.49, p = 0.001

Correlations between serum and tissue analyte levels		
	FD Muscle	FD Tendon
Serum IL-6	IL-6: r = 0.36, p = 0.03	IL-6: r = 0.55, p = 0.0004
Serum TNF-α	TNF-α: r = 0.54, p = 0.0005	TNF-α: r = 0.28, p = 0.04
Serum TGFB1	TGFB1: ns	TGFB1: r = 0.65, p = 0.006
Serum CTGF	CTGF: r = 0.71*, p = 0.04	CTGF#f: r = 0.75, p = 0.03
Serum MMP2	MMP2: ns	MMP2: r = 0.57, p = 0.01

FD = flexor digitorum; n.s. = not significant; n.t. = not tested;

#f number of CTGF-immunopositive fibroblasts in epitendon were used for this correlation.

## Discussion

We hypothesized that grip strength declines would be associated with forearm musculotendinous inflammatory and fibrotic changes with long-term overuse activity at a moderate task level. We observed persistent and progressive declines in forearm grip strength in reach limbs of HRLF rats, but complete recovery with rest in TR24 rats, and a partial recovery in the support limbs. Persistent low-grade increases of inflammatory cytokines were observed in flexor digitorum tissues after the initial training period and throughout task performance, with higher responses in the tendons than in the muscles, and significantly higher responses in the reach limb tissues than in the support limb tissues. The cytokine profile varied, dependent on tissue type, time of task performance, and cytokine type (with, for example, a key anti-inflammatory cytokine, IL-10, increased only in 24-week HRLF rats, but pro-inflammatory cytokines showing earlier and more cyclical type responses). Two fibrogenic proteins (TGFB1 and CTGF) and a collagenolytic protein (MMP2) increased in reach limb muscles and/or tendons of 18- and 24-week HRLF rats, temporally matching epitendon fibrosis in the reach limb. Our hypothesis was supported in that grip strength of the reach limbs correlated inversely with flexor digitorum tissue levels of IL-6, TNF-α, TGFB1, CTGF and MMP2 from these same limbs. We also sought to identify serum biomarkers indicative of underlying tissue processes, and hypothesized that at least one serum biomarker of each process would be increased. Serum levels of TNF-α and IL-6 temporally matched and correlated with tissue increases. We also shown here for the first time that four fibrogenic proteins, TGFB1, CTGF, PDGFab and PDGFbb, hydroxyproline, as well as MMP2, a degradative collagenase, increased in serum with performance a HRLF task for weeks 18 or 24, concomitant with peritendon hyperplasia, increased tissue TGFB1, CTGF, hydroxyproline, and MMP2. Lastly, grip strength correlated inversely with serum levels of IL-6, TNF-α, TGFB1 and MMP2, and showed a strong trend towards correlating with serum CTGF levels.

This is our first study examining grip strength declines in rats performing repetitive tasks for more than 12 weeks. The data demonstrate that there are significant effects of training on grip strength in the reach limbs that persisted and worsened in rats that continued to perform the HRLF task for 24 weeks. In a recent study, declines in grip strength were also present, in the reach limbs, immediately after training in rats that had just learned a high force task, but not a negligible force task, indicating an exposure dependent effect of training on grip strength [47]. Apparently, training for 10 min/day, 5 days/week, for 4–6 weeks to learn moderate (e.g. this HRLF task) or high force repetitive tasks impacts grip strength in the preferred reach limbs negatively. In TR24 (TR+Rest) rats, grip strength declines resolved by 18 weeks of rest, suggestive of tissue healing as a consequence of the rest. We observed only a partial recovery of grip strength in young adult TR+Rest rats in a recent 12-week study, in which the trained only rats rested for only 12 weeks [46]. These findings suggest that long rest periods are needed for complete recovery of grip strength after the initial training period.

We also observed declines in grip strength in the contralateral, support limbs of these HRLF rats after training and through week 18. The declines in the support limb are most likely due to its use for support against the operant chamber wall during the task, as previously examined in rats performing a high repetition high force task for 12 weeks [48]. The declines in grip strength in the support limbs of rats performing a high repetition high force in our prior study [48] was considerably more than observed in this study, in which rats are performing a high repetition low force task, suggesting that tissues in the high force study were nearing their threshold for failure [54]. However, in this study examining the effects of a low force task, the recovery of grip strength in the HRLF support limbs by week 18 is likely due to adaptation of tissues to the demands of providing support. In contrast, the persistent and progressive grip strength declines in the reach limbs of HRLF rats indicate that tissues in that limb are not adapting to the moderate demands of this repetitive task.

We observed a low-grade, cyclical, tissue inflammatory response in musculotendinous tissues with performance of this HRLF task for 24 weeks, extending our past shorter studies of 8 to 12 weeks examining the effects of this HRLF task and a related high repetition negligible force (HRNF) task [42], [44], [47], [55]. What we did not expect, but observed, were differential cytokine responses in flexor digitorum tissues over time. For example, tendon responses were greater than those in muscles, and preferred reach limb tissues were greater than in the support limb tissues. The specific cytokines examined also varied in their response profiles. IL-1β, TNF-α and IL-6 increased after training, bilaterally, since the rats tend to not show limb dominance during training. TNF-α was resolved, bilaterally, by week 18 in HRLF rat tendons and muscles. IL-6 had resolved only in the support limb by week 18, also it was still elevated in reach limb tendons of HRLF rats in week 18, as was but IL-1α and IL-1β. By week 24, TNF-α had increased again in HRLF reach limb tissues, compared to control and TR24 (TR+Rest) rats; IL-6 was increased in reach limb HRLF tissues, compared to control and TR24 rats; and IL-10 was increased in muscles, compared to TR+Rest rats. The inflammatory cytokine response after the training period is likely due to the onset of an injury-induced cytokine response [56]. The resolution of inflammation in TR24 (TR+Rest) rats and in the HRLF support limbs by week 18 is likely due to tissue repair as a consequence of rest and adaptation, respectively, as is the partial resolution in week 18 in the reach limbs. This is supported by our prior results showing only a transient inflammatory response in tissues of rats performing lower demand tasks [42], [45]. However, the reappearance of the tissue inflammatory response and the increased IL-10 (an anti-inflammatory cytokine) in week 24 in the reach limbs, and partially in the support limbs (IL-6), suggests that tissue adaptation processes are not keeping pace with tissue injury or degradative processes. The cyclical inflammatory episodes are consistent with an overexertion theory of MSD development [57], which postulates that when tissue exposure level remains below a critical threshold, inflammatory and repair processes occur that are successful in resolving tissue disruption and restoring normal tissue tolerance through healing. When tissue exposure exceeds this critical threshold, persistent or cyclical inflammatory changes and incomplete tissue healing results in reduced tissue tolerance and function [57] (the latter indicated by the reduced grip strength in this study).

We also observed a serum inflammatory response with performance of the HRLF task for 18 to 24 weeks, extending our past shorter studies of 8 to 12 weeks with this task and a related high repetition negligible force (HRNF) task [42], [43], [46], with temporal differences across weeks of task performance. The variable increase after training indicates that the 10 min/day, 5 days/week, 4–5 week training period in which rats are learning this moderate level tasks is not enough to induce significant increases of inflammatory cytokines in blood, matching findings in prior studies from our lab examining the effects of learning low force tasks [42], [46]. However, continued performance of the HRLF task induced increased serum MIP2 and interferon gamma by week 6 [46], a resolution of the serum response by week 12 (to be specific, no increase of IL-1α, TNF-α, MIP2, IL-6 or IL-10) [46], but reappearance in weeks 18 (IL-1α and IL-12) and 24 (TNF-α and IL-6) in this current study. The increase of IL-10 in HRLF week 18 may be one reason for the decline of IL-1α in HRLF week 24, since IL-10 plays a key role in limiting immune responses [58]. IL-6 also has anti-inflammatory properties, as discussed further below. The increase of TNF-α in week 24 may be due to the increase of IL-12 in week 18, a known inducer of TNF-α in macrophages [59]. We have shown that macrophages increase in flexor digitorum tissues with prolonged HRLF task performance [55].

The serum inflammatory cytokines in patients with upper extremity MSDs diagnoses (patients with signs and symptoms of carpal tunnel syndrome, rotator cuff tendinitis, and medial/lateral epicondylitis) is also varied temporally. In patients with symptoms of 1 month or less in duration, serum levels of soluble IL-1RII and IL-18 increase (but not TNF-α, IL-1α, IL-1β or IL-6). In patients with symptoms of 3 months in duration, serum levels of TNF-α, IL-1β and IL-6 increase [2]. Although the duration of symptoms were not reported in a third study, video terminal operators with mild to moderate functional impairments and localized pain (indicative of having worked as video terminal operators for at least long enough to develop mild to moderate work-related musculoskeletal disorders) have increased serum IL-6 and TNF-α, compared to healthy controls [4]. The members of the IL-1 family appear to be functioning more as early onset responders and biomarkers, with TNF-α and IL-6 as later phase biomarkers of injury and inflammation.

However, increased IL-6 in tissues and serum is subject to multiple interpretations. Many forms of exercise can induce increased tissue and serum inflammatory cytokines, including IL-6, although these increases are usually rapid in nature [60], [61]. In this study, it is not likely that the observed inflammatory cytokines increases are exercise-induced, since we waited 36 hours after the last task session to collect tissues and serum. IL-6 has been reported to have both pro- and anti-inflammatory actions; therefore, it may be acting in an anti-inflammatory mode with IL-10 [20], [60], although since it increased in week 24, the end of our experimental paradigm, we are not able to speculate on that possible function here. IL-6 is also a fibrotic protein and up-regulates CTGF production by fibroblasts in vitro [19]. This is of interest because tissues collected from humans with upper extremity work-related musculoskeletal disorders show the presence of fibrosis and its mediators, including IL-6 and TGFB1 [11], [23], [62]. IL-1β and TNF-α have also been deemed pro-fibrotic, due to their mitogenic and chemotactic effects on fibroblasts and induction of TGFB1 [14], [15], [16], [17], [18]. We postulate that IL-1β, TNF-α IL-6 increase at points of tissue injury or worsening tissue injury (such as after training and then in week 24 after a failed resolution stage due to continued task performance), and that their persistent increase is contributing to the later appearing fibrotic responses.

As stated in the introduction, TGFB1 and CTGF are sensitive serum biomarkers of fibrogenic diseases [26], [30]. This is the first time, to our knowledge, that serum increases of either have been linked to work-related musculoskeletal disorders. Several studies have shown that TGFB1 and CTGF increase in tissues under conditions of overload or injury [63], [64], [65]. Increases of TGFB1, CTGF and IL-6, have been linked to the pathogenesis of tissue fibrosis [66], [67], [68], [69]. Stauber and colleagues reported elevated levels of TGF-beta1 precursor proteins in strain-injured skeletal muscles 48 h after injury [65], although like this study, no increase in TGFB1 active subunit (12.5 kDa), postulating potential rapid clearance of the active form from the muscle. CTGF is the downstream mediator of TGFB1 and is produced by multiple cell types [70], [71], [72]. TGFB1-induced CTGF expression leads to fibroblast proliferation and extracellular matrix deposition [72]. However, CTGF production is also regulated by TNF-α [47], [73], [74], which is interesting since we observe increases of each in reach limb tissues and serum in this study. Furthermore, CTGF stimulation increases IL6, IL-1 and TNF-α mRNA levels in human tendon fibroblasts, suggestive of an autocrine/paracrine feedback loop between these fibrogenic proteins [66]. In our model, we have previously reported increased CTGF in peripheral nerves, muscles, and epitendons of involved forearm and shoulder tendons with prolonged performance of low to high demand repetitive tasks [47], [48], [51], [55], [71]. We recently reported that a two-week treatment with an anti-rat TNF- α monoclonal antibody decreased CTGF and collagen extracellular matrix production (although TGFB1 levels did not alter) [47]. The fibrotic changes observed in this current study were mild, with no increase in serum PINP, modest increases of CTGF in serum and reach limb muscles and tendons, although significant increases of hydroxyproline in both serum and reach limb muscles. The latter is suggestive of collagen accumulation in tissues, and has been shown to increase in fibrotic diaphragm muscles of the mdx mouse, a model for Duchenne dystrophy [89]. Increased serum hydroxyproline has been indicated as a biochemical predictor of liver fibrosis, especially when combined with other serum biomarkers of collagen accumulation and turnover [90], [91] that we are observing the onset of fibrosis in these 18-week HRLF rats

It has been suggested that inhibition of CTGF activity could be a suitable target for prevention of fibrotic disorders [75], [76]. In this study we have demonstrated significant up-regulation of CTGF expression that correlates with the fibrotic changes observed in the HFLF model, but we have not established a causal relationship these two findings. Additional studies are underway to determine whether elevated CTGF is an essential mediator of these fibrotic events. If so, then one could envision CTGF as a potential therapeutic target to prevent the fibrosis and reduced function that occurs as a consequence of overuse.

We also observed an increase in two subtypes of PDGF. PDGF is a repair cytokine synthesized by numerous cell types, including platelets, macrophages, fibroblasts and osteoblasts, and known to promote skeletogenesis, organogenesis, angiogenesis and wound healing [77]–[79]. While it is clear that PDGF subtypes are not increased in reach limb tendons in our model, their increase in serum indicate that another tissue is responding to the repetitive loading with increased PDGF production. Future studies should focus on determining its sources, such as in repetitively loaded bones.

An increase in the collagenolytic gelatinase, MMP2, was evident in both serum and forearm tendons of reach limbs of 18- and 24-week HRLF rats. Hirata and colleagues have previously shown increased MMP2 in flexor tendosynovial tissues collected from patients with carpal tunnel syndrome, with higher MMP2 activity in patients with a sudden exacerbation of symptoms [24]. MMP2 also increases in tendons after vertical jumping and treadmill running, with greater increase in distal tendon regions (the same region examined in this study) than in proximal tendon regions [80]. MMPs are involved in collagen catabolism, and show increased activity with high or low mechanical loading, particularly when sustained over prolonged periods or during periods of tendon repair after injury [80], [81], [82]. Increases of both TGFB1 and CTGF lead to an increase in MMP2 expression and fibroblast activity *in vivo* [83]. The observed increase of MMP2 in reach limb tendons support the presence of tendon degenerative processes occurring as a result of long-term performance of this moderate demand task. MMP2 has also been shown to increase the conversion of TGFB1 precursor subunits to the active form of TGFB1 [84]. The overall increase of MMP2 in reach limb tendons and serum suggest that with continued task performance, that more TGF-beta1 precursor proteins will be converted to its active form, a conversion that is likely to lead to continued task-induced fibrosis.

Lastly, these data are suggestive of multiple contributors to the observed decline grip strength in the reach limbs. Increased muscle inflammation, such as increased muscle TNF-α, is known to reduce forearm grip strength [85], [86], [87]. In this study, and in two prior studies, we have observed correlations between grip strength declines and tissue/serum inflammatory cytokines [42], [44], [46]. Patients with signs and symptoms of musculoskeletal disorders (including reduced grip strength) also show increased serum inflammatory cytokines that correlate with functional impairments [2], [3], [4]. For example, serum levels of TNF-α, IL-1β and IL-6 correlate positively with presence of musculoskeletal disorders symptoms in patients, and TNF-α levels are even predictive of symptom severity [2]. The temporal association between grip strength declines and increased TGFB1, CTGF, MMP2, and fibrotic tendon changes suggest that even fairly early tissue fibrosis is contributing to motor declines. This is also supported by prior work from our lab and others showing increased fibrogenic proteins, musculotendinous fibrosis and degenerative processes with prolonged exercise or repetitive loading in animal models [35], [37], [48], [51], [55], [63], [64], [65], [71], [88].

In conclusion, these findings show that even moderate demand tasks can induce tissue degradative changes if the work is performed for long periods of time. The temporal association of grip strength declines with both low-grade tissue inflammatory cytokine response and increased fibrotic/degradative proteins support a contribution from each process to functional motor declines occurring with overuse. Although further studies on humans are needed, these findings suggest that serum levels of TGFB1, CTGF and MMP2, and perhaps even hydroxyproline, may serve as serum biomarkers of underlying tissue fibrogenic changes in this rat model of work-related musculoskeletal disorders, and that serum IL-6 may be a biomarker of either musculotendinous inflammatory or fibrotic responses.
